# Associations among stress perception, clinical symptoms, and hippocampal subfield volumes in first-episode patients with schizophrenia

**DOI:** 10.3389/fpsyt.2026.1885091

**Published:** 2026-07-23

**Authors:** Ting Xie, Zhaofan Liu, Ping Zhang, Junchao Huang, Yanfang Zhou, Tianyi Zhu, Xiaoying Wang, Jinghui Tong, Mengzhuang Gou, Wenjin Chen, Yi Yin, Ting Yu, Wei Li, Song Chen, Yanli Li, Xingguang Luo, Zhiren Wang, Li Tian, Fude Yang, Chiang-Shan R. Li, Yunlong Tan

**Affiliations:** 1 Peking University HuiLongGuan Clinical Medical School, Beijing Huilongguan Hospital, Beijing, China; 2 Department of Psychiatry, Yale University School of Medicine, New Haven, CT, United States; 3 Department of Psychology and Logopedics, Faculty of Medicine, University of Helsinki, Helsinki, Finland

**Keywords:** clinical symptoms, first-episode schizophrenia, hippocampal volume, schizophrenia, stress perception

## Abstract

**Background:**

Hippocampal neurogenesis shapes adaptation and improves responses to stress. Patients with schizophrenia show marked functional impairment and abnormal stress sensitivity. However, it remains unclear how structural abnormalities in specific hippocampal subregions are related to altered perceived stress and clinical symptoms in schizophrenia.

**Methods:**

We recruited 97 first-episode patients with schizophrenia (FEPS) and 47 healthy controls (HC). Perceived stress and psychopathology were assessed using the Perceived Stress Scale (PSS) and the Positive and Negative Syndrome Scale (PANSS), respectively. Structural MRI was acquired on a 3.0-T scanner, and hippocampal subregions were segmented using validated, standardized protocols. Left and right subregional volumes were summed to obtain calculate bilateral volumes.

**Results:**

Compared to HC, the FEPS group showed higher perceived stress, reduced fimbria volumes, and increased hippocampal tail volumes after adjustment for intracranial volume, age, sex, and education. In HC, several hippocampal subregion volumes were positively correlated with stress perception; however, these associations were absent or disrupted in FEPS. In FEPS, stress perception was positively associated with positive and anxiety/depression symptoms. Additionally, specific hippocampal subfield volume was positively associated with negative symptoms.

**Conclusions:**

FEPS was characterized by aberrant perceived stress, altered hippocampal subregional volumes, and disrupted links between stress perception and hippocampal structure. The interrelations among stress, clinical symptoms, and hippocampal subfields suggest altered psychological and neurobiological processes underlying stress regulation processes in FEPS.

## Introduction

Schizophrenia is a severe mental disorder that often leads to substantial functional impairment (1) and disease burden (2). The pathophysiology of schizophrenia is widely considered to involve a complex interactions between genetic and environmental factors (3). Among the environmental influences, psychosocial stress has consistently been identified as a critical risk factor for both the onset and chronic course of schizophrenia (4, 5). Accumulating evidence suggests that exposure to stress can lead to dysregulated dopaminergic neurotransmission (6, 7), providing a neurobiological link between stress and psychotic symptoms.

Perceived stress reflects how stressors are appraised and experienced by individuals. Previous studies have associated elevated perceived stress with psychosis-like experiences, which are subthreshold phenomena that may precede the onset of schizophrenia (8, 9). Moreover, perceived stress has been shown to mediate the relationship between social functioning and quality of life in individuals at ultra-high risk for psychosis (10). In patients with first-episode schizophrenia, higher levels of perceived stress have also been consistently associated with depression (11, 12), and suicidality (13).

Stress dysregulation involves key brain regions of the corticolimbic system (14). Neuroimaging studies have reported modest associations between perceived stress and gray matter volumes of the hippocampus and amygdala (both *r*’s < 0.3) (15). As a primary target of stress hormones (16, 17), the hippocampal circuitry is central to stress-related structural and functional brain plasticity (18). Importantly, hippocampal neurogenesis plays a key role in shaping individual stress experiences and coping responses (19).

The hippocampus also plays a pivotal role in the pathophysiology of schizophrenia (20). Hippocampal morphometric alterations have been observed across different phases of schizophrenia and are thought to reflect dynamic neural plasticity (21, 22).Notably, hippocampal pathology in schizophrenia is generally not considered to be strictly neurodegenerative in nature (23). Hippocampal dysfunction, particularly the loss of parvalbumin interneurons, alters downstream dopaminergic signaling (24). In addition, impairments in dentate gyrus-specific mnemonic functions may increase illusory pattern completion and reduce discrimination between present and past experiences, thereby contributing to psychotic symptom formation (25).

Thus, elevated perceived stress and hippocampal dysfunction are both implicated in schizophrenia; however, how hippocampal dysfunction relates to perceived stress, and clinical symptoms in schizophrenia remains poorly understood. The present study aims to address this gap by investigating perceived stress, structural alterations in hippocampal subregions, and their associations with clinical symptoms in first-episode patients with schizophrenia (FEPS), a population with minimal exposure to antipsychotic medication. We compared stress perception and hippocampal subregional volumes between patients and healthy controls and assessed whether the associations between stress perception and hippocampal volumes were altered in schizophrenia. In addition, exploratory analyses were performed to further characterize the interrelationships among hippocampal subregional volumes, perceived stress and clinical symptoms in FEPS.

## Methods

### Participants

Initially, 120 first-episode patients with schizophrenia (FEPS) and 47 healthy controls (HC) were recruited for this study. After excluding 23 FEPS who did not undergo MRI scanning, a final sample of 144 participants (97 FEPS and 47 HC) was included in the current analyses. Patients were recruited from Beijing Huilongguan Hospital. Inclusion criteria for patients were as follows: (1) a diagnosis of schizophrenia according to the Diagnostic and Statistical Manual of Mental Disorders, the 5^th^ Edition (DSM-5), confirmed by structured clinical interviews conducted by two trained psychiatrists; (2) age between 16 and 45 years; (3) total illness duration not exceeding 3 years; and (4) duration of antipsychotic treatment not exceeding 2 weeks at the time of assessment. Exclusion criteria were as follows: (1) comorbid intellectual disability; (2) serious physical or organic brain diseases; (3) substance (except nicotine) use disorders; (4) pregnancy or lactation; and (5) recent injury or allergic episodes.

HC were recruited from the local community and were matched to patients by age and sex. Exclusion criteria for the HC group included a personal history of any psychiatric disorders, as confirmed by formal psychiatric interviews. All participants underwent a comprehensive medical history interview, physical examination, laboratory testing (including urine and blood screening), and electrocardiogram assessment, and no relevant neurological or systemic illnesses were identified.

The study was conducted in accordance with the Declaration of Helsinki of 1975, as revised in 2013. It was approved by the Medical Ethics Committee of Beijing Huilongguan Hospital (registration number: 2022-11-Ke, and the approval date: February 18, 2022). All participants have signed written informed consent prior to the study participation, and the privacy rights of human subjects were observed throughout the study.

### Clinical measures

Perceived stress was assessed using the Perceived Stress Scale (PSS), developed by Cohen et al. (26), which is one of the most widely used instruments for evaluating perceived stress. The PSS quantifies the extent to which individuals perceived their lives as stressful and uncontrollable during the past month. It contains 14 items rated on a 5-point scale from ‘never’ (0 points) to ‘almost always’ (4 points). Seven items assess the perceived stress (items 1, 2, 3, 8, 11, 12, and 14), whereas the other seven items assess perceived coping or control (items 4, 5, 6, 7, 9, 10, and 13). The PSS was self-administered under the supervision and guidance of trained clinicians.

Psychopathology was evaluated using the Positive and Negative Syndrome Scale (PANSS). Two psychiatrists independently administered the PANSS on the same day the MRI data were collected. Both raters received standardized training in PANSS on the administration and scoring before the study to ensure reliability. The intraclass correlation coefficient (ICC) for PANSS total scores exceeded 0.75, indicating good inter-rater reliability. Two trained psychologists administered the PANSS and concurrently supervised the completion of the PSS.

### MRI protocol and data processing

MRI data were acquired at the MRI Research Center of Beijing Huilongguan Hospital using a 3.0-T Prisma MRI scanner (Siemens, Erlangen, Germany) and a 64-channel head coil. Structural T1-weighted images were acquired using a sagittal three-dimensional magnetization-prepared rapid gradient echo (MP-RAGE) sequence, with the following scanning parameters: repetition time (TR) = 2530 ms, echo time (TE) = 2.98 ms, flip angle (FA) = 7°, matrix size = 256×224, field of view (FOV) = 256×224 mm, inversion time (TI) = 1100 ms, and slice thickness/gap = 1/0 mm.

Hippocampal volume was computed using FreeSurfer software (27) version 6.0. For each participant, bilateral hippocampi were segmented into the following subregions: hippocampal tail, subiculum, CA1, hippocampal fissure, presubiculum, parasubiculum, molecular layer, granule cell and molecular layer of the dentate gyrus (GC–ML–DG), CA2/3, CA4, fimbria, and hippocampus–amygdala transition area (HATA. Subregional volumes were extracted separately for the left and right hemispheres. All segmentations were visually inspected for quality control by trained raters who were blinded to diagnostic status. Intracranial volume (ICV) was estimated using FreeSurfer’s automated procedure and was included as a covariate in subsequent analyses to account for individual differences in head size.

The subregional atlas was based on high-resolution (0.13 mm) ex vivo MRI data obtained from a 7.0-T scanner. Quality control followed the ENIGMA protocol and consisted of two steps: (1) visual inspection by overlaying hippocampal segmentation images onto anatomical images; and (2) exclusion of volumetric outliers, defined as values exceeding five standard deviations from the mean. No subjects were excluded based on these quality control procedures.

### Statistical analysis

Group differences in demographic and clinical variables between patients and HC were examined using appropriate parametric or non-parametric tests. Continuous variables were initially assessed for normality and homogeneity of variance. When the assumption of homogeneity of variance was met, independent-samples *t* tests were applied; otherwise, Welch’s *t* tests were used. For variables violating normality assumptions, the Mann-Whitney U test was employed. Categorical variables, including sex, were compared using the chi-square (χ²) test. Group differences in hippocampal subfield volumes were assessed using analysis of covariance (ANCOVA), with intracranial volume (ICV), age, sex, and years of education and chlorpromazine (CPZ) dose equivalents included as covariates to control for potential confounding effects. All statistical tests for group comparisons were two-tailed. Statistical analyses were conducted in Python (version 3.8.15) using the scipy.stats library.

Associations among perceived stress, hippocampal subfield volumes, and clinical symptom measures were examined using partial correlation analyses. To evaluate the potential confounding effect of educational attainment on stress-related associations, two partial correlation models were computed: Model 1 adjusted for age, sex, and CPZ dose; and Model 2 additionally adjusted for years of education. Correlation coefficients (*r*) and corresponding two-tailed *p* values were calculated to quantify the strength and significance of linear relationships. To account for multiple comparisons, false discovery rate (FDR) correction was applied independently across the subfield dimensions for each clinical symptom domain. Statistical analyses were conducted in Python (version 3.8.15) using the scipy.stats and pingouin modules.

## Results

### Demographic, clinical characteristics and hippocampal volumes of patients and healthy controls

**Table 1** summarizes the demographic and clinical characteristics of the first-episode patients with schizophrenia (FEPS) and the heathy controls (HC). No significant differences were observed between the two groups in age or sex distribution. However, the HC group had significantly higher education compared to the FEPS group. In terms of stress-related measures, the FEPS group demonstrated significant alterations in stress processing. Specifically, compared with the HC, FEPS showed significantly higher perceived stress scores (*t* = 5.23, *p* < 0.001) and lower level of stress coping (*t* = -4.67, *p* < 0.001) (**Table 1**). There were 16 patients who were drug-naïve (received no antipsychotic medication); the other patients received second-generation antipsychotic medications. The chlorpromazine equivalents are presented in **Table 1**, and specific drug types are detailed in **Supplementary Table 1**.

**Table 1:** Demographic data and clinical symptoms in the FEPS and the HC.

Characteristic	FEPS (n=97)	HC (n=47)	*t/χ2*	*p*
Sex (M/F)	52/45	21/26	0.68	0.40
Age (years)	27.7 (9.0)	29.6 (7.1)	-1.25	0.21
Education (years)	12.4 (3.4)	13.9 (2.6)	-2.9	0.004
Age of illness onset (years)	26.2 (7.3)	/	/	/
Course of disease (months)	13.1 (16.3)	/	/	/
Number of drug-naïve patients	16	/	/	/
Medications, mg (chlorpromazine equivalents)	304.1 (150.9)	/	/	/
PANSS total Score	75.8 (12.0)	/	/	/
Positive	22.5 (4.4)	/	/	/
Negative	15.1 (5.6)	/	/	/
Disorganization	16.6 (4.6)	/	/	/
Hostility/Excitement	11.9 (4.7)	/	/	/
Anxiety/Depression	9.7 (4.0)	/	/	/
Total PSS score	27.8 (9.8)	18.7 (7.0)	6.39	p<0.001
Stress Perception	16.1(6.0)	11.4 (4.1)	5.23	p<0.001
Stress Coping	11.8 (6.0)	16.5 (4.9)	-4.67	p<0.001

PANSS, Positive and Negative Syndrome Scale; PSS: Perceived Stress Scale.

Regarding hippocampal morphology, no significant group differences were observed in total hippocampal volume between the FEPS and HC groups (all *p*’s > 0.1; **Table 2**). However, selective alterations were identified at the subregional level were identified. Compared with HC, the FEPS group showed significantly reduced volumes in fimbria volumes and increased volumes in hippocampal tail volumes after controlling for intracranial volume (ICV), age, sex, and education (all p’s < 0.05, FDR corrected). No other hippocampal subregions exhibited significant group differences after correction for multiple comparisons.

**Table 2:** Comparison of hippocampal subfield volumes between FEPS and HC groups.

Variables	FEPS (n=97)	HC (n=47)	*t/F*	*p*
Intracranial Volume	1.54*10^6^	1.56*10^6^	-0.70	0.48
hippocampal subfield volumes (mm³)				
Hippocampal tail	1141.46 (136.37)	1076.12 (120.38)	11.262	0.007
Subiculum	893.59 (84.99)	918.28 (85.73)	0.048	0.994
CA1	1236.39 (133.48)	1260.67 (122.79)	1.556	0.591
Hippocampal fissure	311.23 (44.19)	293.39 (34.84)	5.799	0.075
Presubiculum	650.56 (85.64)	632.08 (72.48)	0.000	0.994
Parasubiculum	119.55 (23.44)	119.99 (24.20)	0.672	0.673
Molecular layer	1131.23 (99.81)	1154.35 (101.67)	0.227	0.917
GCMLDG	579.60 (51.92)	599.44 (62.24)	0.002	0.994
CA2/3	386.25 (48.79)	390.12 (57.89)	1.438	0.591
CA4	494.89 (44.37)	509.59 (53.35)	0.004	0.994
Fimbria	169.75 (43.45)	221.93 (32.86)	11.970	0.007
HATA	110.73 (14.23)	116.82 (14.80)	1.031	0.591
Total hippocampal volume	6914.00 (587.26)	6999.39 (609.96)	1.003	0.591

For ICV comparison between groups: an independent samples t-test was conducted after Levene’s test for homogeneity of variance, and Welch’s t-test was used when variances were unequal. For hippocampal subfield volume comparisons: analysis of covariance (ANCOVA) was performed with age, sex, years of education years and ICV as covariates to control for their confounding effects Multiple comparisons were corrected using the false discovery rate (FDR; Benjamini-Hochberg) method with a significance level of α = 0.05. Thus, the statistics reported are F-values and FDR-corrected p-values for each subfield volume.

### Disrupted associations between stress perception and hippocampal volumes in the FEPS

**Figure 1:**
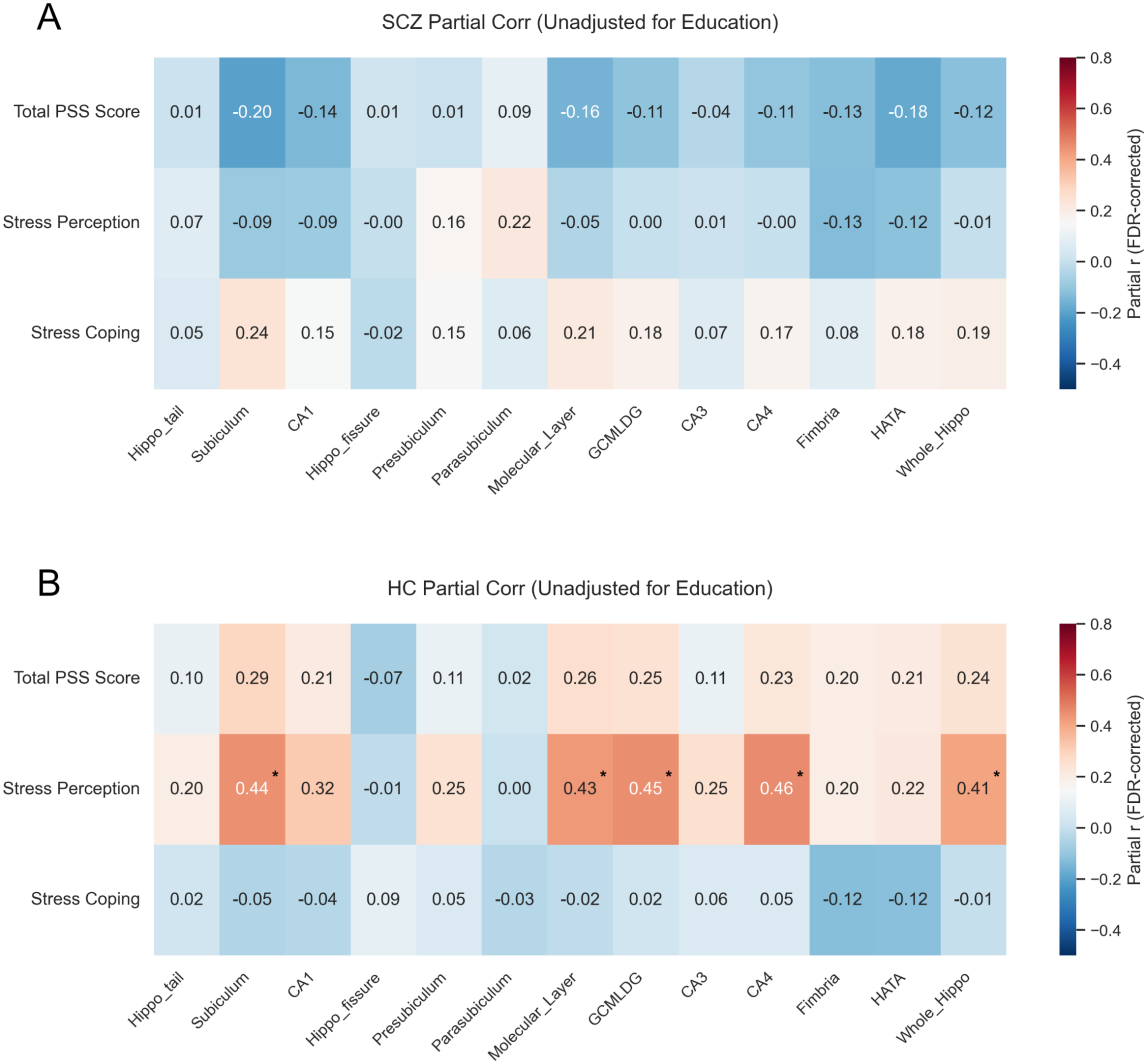
Correlation of stress coping and subdivision hippocampus volume in the FEPS and HC groups. **(A)** Partial correlations and their corresponding p-values in FEPS; **(B)** Partial correlations and corresponding *p*-values in HC. Symbols: * denotes 0.01 < *p* < 0.05 after FDR correction within each group.

Beyond group differences in stress-process and hippcampal subfield volumes, we investigated the associations between perceived stress and hippocampal subfield volumes. Partial correlations between total PSS score, stress perception and stress coping and hippocampal volumes, after adjusting for age, sex and chlorpromazine equivalents in the FEPS and for age and sex in HC, are illustrated in **Figure 1**.

In HC, stress perception was significantly associated with several hippocampal volumes, including the subiculum (*r* = 0.44, *p* < 0.05), molecular layer (*r* = 0.43, *p* < 0.05), GC-ML-DG (*r* = 0.45, *p* < 0.05), CA4 (*r* = 0.46, *p* < 0.05), and total hippocampus (*r* = 0.41, *p* < 0.05). In contrast, these associations disappeared in the FEPS group, indicating a disrupted relationship between perceived stress and hippocampal subfield volumes in FEPS. Furthermore, partial correlations that additionally included education as a covariate were conducted (**Supplementary Figure 1**). Although the aforementioned associations did not survive FDR correction in these models, the pattern of positive correlation remained more evident in HC compared to FEPS, highlighting the potential confounding or mediating role of educational attainment.

### Association of stress perception and hippocampal subfield volumes with the clinical symptoms in FEPS

**Figure 2:**
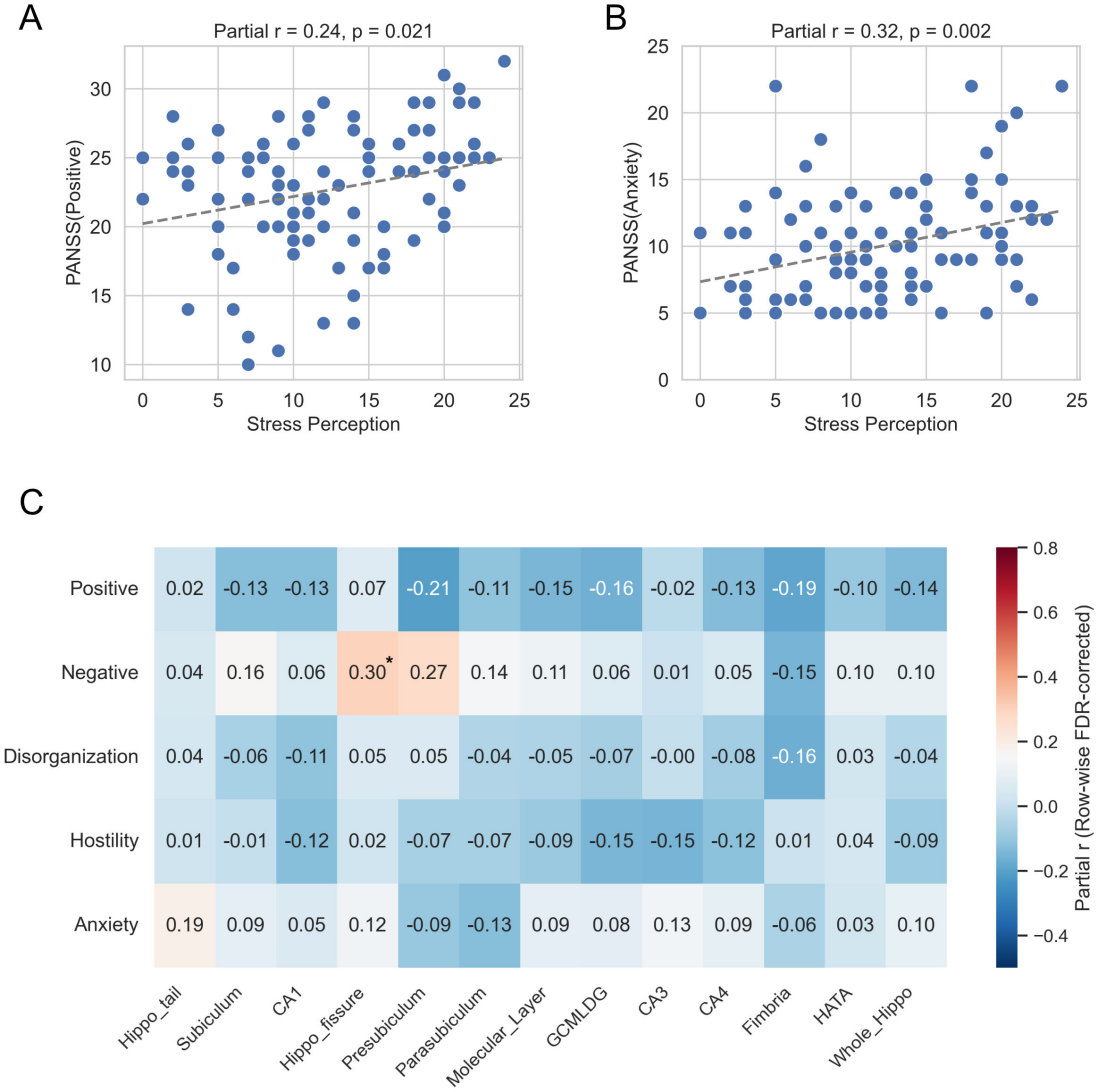
Associations of stress perception and hippcampal subfield hippocampus volumes with clinical symptoms in FEPS. **(A)** Association between stress perception with PANSS positive symptoms. **(B)** Association between stress perception and PANSS anxiety/depression symptoms. **(C)** Partial correlations and corresponding *p* values between clinical symptom dimensions and hippocampal subfield volumes (* denotes 0.01 < *p* < 0.05 after FDR correction).

Having demonstrated that the association between perceived stress and hippocampal subregional volumes was disrupted in the FEPS, we further investigated whether these variables were correlated with clinical symptoms in FEPS. Stress perception was positively correlated with the PANSS positive symptoms (*r* = 0.24, *p* = 0.02, **Figure 2A**), and anxiety/depression symptoms (*r* = 0.32, *p* = 0.002, **Figure 2B**). No significant correlations were observed between stress coping scores and any dimension of the PANSS symptom dimension.

Hippocampal subfields exhibited distinct and region-specific associations with symptom dimensions in the FEPS (**Figure 2C**). Negative symptom severity was positively correlated with volumes of hippocampal fissure volume after controlling for the age, sex, education and medication dose (*r* = 0.30, *p* < 0.05, FDR-corrected).

## Discussion

This study comprehensively examined the relationship between subjective perceived stress and hippocampal subregional volumes in first-episode patients with schizophrenia (FEPS). Our findings indicate that the FEPS exhibited both altered perceived stress and structural abnormalities in specific hippocampal subregions, accompanied by a marked disruption in the association between perceived stress and hippocampal morphology that was observed in healthy controls (HC). Furthermore, exploratory analyses indicated that hippocampal subregional volumes, perceived stress and symptom dimensions were interrelated in the FEPS. Together, these results suggest a disrupted neuropsychological pathway linking hippocampal subregional structure, stress perception, and symptom expression in the FEPS, providing new insights into the neural mechanisms underlying stress dysregulation in the early stage of schizophrenia.

The FEPS showed an abnormal pattern of stress processing, characterized by higher perceived stress and reduced stress coping compared with HC, which is consistent with previous studies (12, 15). These findings align with the stress-vulnerability model of schizophrenia, which proposes that abnormalities in stress appraisal and regulation emerge early in the course of the illness (28, 29). This pattern suggests that the FEPS not only experience stress more intensely but also exhibit diminished stress-buffering capacity. Elevated stress perception may reflect heightened vigilance and sensitivity to environmental stressors, whereas reduced stress coping may indicate impaired stress dampening or emotional numbing (30). Over time, exposure to environmental stressors, such as trauma, may increase the risk of psychotic episodes and hospitalization through stress sensitization, whereby repeated or severe stress amplifies vulnerability to psychosis (31).

Beyond altered stress perception and coping, we observed structural alterations in hippocampal subregions in the FEPS. Previous studies have consistently reported hippocampal subfield volume reductions in schizophrenia (32–34). However, the present study showed that hippocampal subregional volumes in FEPS were not uniformly reduced, consistent with a previous study (35). Specifically, increased hippocampal tail volumes and reduced the hippocampal fissure volumes were observed. These findings may reflect the early stage of illness, suggesting that hippocampal alterations in the FEPS may not yet exhibit the pronounced degenerative changes reported in chronic schizophrenia (36).

Rather than indicating neurodegeneration, the observed hippocampal tail enlargement may reflect developmental delay, stress-related plasticity, or compensatory structural changes during the early phase of the disorder (23). However, this interpretation should be made with caution. The hippocampal tail is anatomically complex, and methodological studies have highlighted that automated segmentation of the tail, particularly in the FreeSurfer version utilized in this study, has lower spatial reliability and is more susceptible to boundary artifacts compared to the hippocampal head or body (37). In addition, we observed reduced fimbria volumes, a finding that has been reported previously, and may be related to early white matter vulnerability or oxidative stress processes in the FEPS, both of which have been implicated in hippocampal circuitry dysfunction (38).

In HC, hippocampal subregional volumes were significantly associated with stress perception, suggesting an adaptive coupling between stress processing and hippocampal structural plasticity, with educational attainment potentially playing a vital role in stress processing. In contrast, this association was largely disrupted in the FEPS, indicating impaired stress-related hippocampal modulation. Stressful experiences are known to trigger the release of glucocorticoids (39), and appropriate levels of cortisol have been shown to support hippocampal plasticity (40). However, excessive or dysregulated stress exposure can impair hippocampal structure, reduce neuroplasticity, and alter cellular composition and white matter integrity, thereby increasing vulnerability to mental illness (40). Moreover, prior evidence indicates that perceptual training can alter the neural plasticity (41, 42); therefore, educational experience may also interact with stress perception. Our findings suggest that this adaptive stress-hippocampal relationship and the protective effect of education may be compromised at an early stage of schizophrenia.

Exploratory analyses further revealed that positive/anxiety symptoms were correlated with stress perception, whereas and negative symptoms were correlated with the hippocampal fissure volume. Because symptoms in schizophrenia can be conceptualized as a network (43–45), associations among negative symptoms, positive/anxiety symptoms, stress perception and hippocampal subfield volumes may reflect interconnected psychological and neurobiological processed in FEPS. These findings highlight the critical role of hippocampal–dopaminergic interactions in schizophrenia (46). The ventral hippocampus, particularly regions encompassing the molecular layer and dentate gyrus (GC-ML-DG), is known to modulate the activity of ventral tegmental area dopamine neurons (47–49), while dopaminergic signaling, in turn, influences hippocampal structure and function (50, 51). This bidirectional hippocampus-dopamine pathway may underlie the observed links among clinical symptoms, hippocampal subregional volumes, and stress dysregulation, further emphasizing the central role of the hippocampus in the pathophysiology of schizophrenia.

Our findings underscore the importance of perceived stress and hippocampal subregional alterations in the early stage of schizophrenia. These results suggest that early interventions targeting stress regulation, such as cognitive-behavioral stress reduction strategies (52), may be beneficial as adjunctive approaches to standard treatment. Given the close links among hippocampal structure, immune regulation, and neuroplasticity, lifestyle-related interventions, including physical exercise (53, 54) and behavioral modification (55), may also support hippocampal health in early psychosis. Moreover, as hippocampal dysfunction may contribute to dopaminergic dysregulation (47, 49), future studies should explore whether non-invasive neuromodulation approaches targeting hippocampal-related circuits could offer additional therapeutic benefits in schizophrenia (56, 57).

## Limitations and future directions

Several limitations should be noted. First, the marked symptom heterogeneity of schizophrenia (58, 59) and its potential interactions with stress perception and hippocampal subregional alterations were not fully examined. Different symptom dimensions may be differentially related to stress perception and hippocampal structure, and future studies with larger samples are needed to explore symptom-specific associations. Second, although patients in this study had minimal exposure to antipsychotic medication, antipsychotics can modulate the hippocampal volume, further work should include larger drug-naïve samples. Third, the cross-sectional design precludes causal inference. Longitudinal studies are required to determine whether alterations in hippocampal subfields precede changes in perceived stress and symptom profiles, or whether the reverse temporal sequence occurs. Finally, because the PSS relies on self-reporting and may be subject to various confounding factors, future studies should incorporate objective biological measurements or specific behavioral tasks to more rigorously evaluate stress reactivity.

## Conclusions

In summary, the present study provides a novel perspective on the pathophysiology of first−episode schizophrenia by delineating how perceived stress, hippocampal subfield structure, and symptom dimensions are interlinked, with particular emphasis on the association between hippocampal subregions and perceived stress via positive symptoms. These findings highlight the potential of stress−related biomarkers and hippocampal subregion metrics as candidates for more personalized intervention strategies in first−episode schizophrenia, including stratification by biological subtypes and stress−sensitivity profiles.

## Data Availability

The original contributions presented in the study are included in the article/**Supplementary Material**. Further inquiries can be directed to the corresponding author.
