# Supplementary material for: Associations among stress perception, clinical symptoms, and hippocampal subfield volumes in first-episode patients with schizophrenia
Source: Front Psychiatry. 2026 Jul 23;17:1885091. doi: 10.3389/fpsyt.2026.1885091 (PMC13444367; doi:10.3389/fpsyt.2026.1885091)
Supplement: Supplementary file 1 [file SupplementaryFile1.docx]

**Supplementary Materials**

**Table S1** Specific medication types used in the FEPS group.

| Medication use | Number of patients |
| --- | --- |
| Drug-naive | 16 |
| Aripiprazole | 14 |
| Paliperidone | 3 |
| Olanzapine | 20 |
| Risperidone | 44 |

**Table S2** Medication effects on hippocampal subfield volumes.

| Hippocampal Subfield | β (Medication state) | p | β(doses) | *p* |
| --- | --- | --- | --- | --- |
| Hippocampal tail | -0.274 | 0.305 | -0.060 | 0.531 |
| Subiculum | -0.322 | 0.230 | -0.082 | 0.398 |
| CA1 | -0.391 | 0.149 | -0.185 | 0.056 |
| Hippocampal fissure | -0.214 | 0.399 | -0.030 | 0.744 |
| **Presubiculum** | 0.000 | 0.999 | 0.199 | 0.033 |
| Parasubiculum | 0.074 | 0.783 | 0.159 | 0.099 |
| Molecular layer | -0.268 | 0.310 | -0.109 | 0.249 |
| GCMLDG | -0.049 | 0.855 | -0.125 | 0.199 |
| CA2/3 | 0.071 | 0.795 | -0.148 | 0.132 |
| CA4 | -0.015 | 0.956 | -0.102 | 0.295 |
| **Fimbria** | -0.517 | 0.059 | -0.284 | 0.004 |
| HATA | -0.242 | 0.357 | -0.032 | 0.737 |
| Whole Hippocampal | -0.285 | 0.259 | -0.104 | 0.253 |

**Figure S1** Partial correlations between stress coping and hippocampal subfield volumes in the FEPS and HC groups, after adjusting for education. (A) Partial correlations and corresponding p-values in the FEPS group. (B) Partial correlations and corresponding p-values in the HC group. All p-values were FDR-corrected within each group.
